# Pattern Recognition Classification and Identification of Trace Organic Pollutants in Ambient Air from Mass Spectra

**DOI:** 10.6028/jres.093.045

**Published:** 1988-06-01

**Authors:** Donald R. Scott, William J. Dunn, S. L. Emery

**Affiliations:** U.S. Environmental Protection Agency, Research Triangle Park, NC 27711; College of Pharmacy University of Illinois at Chicago Chicago, IL 60612

## Introduction

The most frequently used method of analysis of trace organic pollutants in ambient air is gas chromatography-mass spectrometry (GC-MS). Presently, target compounds in air monitoring samples are identified from gas chromatographic retention times and a combined forward-reverse search of mass spectral reference spectra. The primary objective of this study is to develop computational pattern recognition procedures for the identification of chemical classes and, if possible, individual compounds from routine GC-MS data files. The additional information on nontarget compounds would supplement compound identifications obtained from the target list. The target set of compounds investigated consisted of 78 substituted benzenes, haloalkanes and haloalkenes.

## Methods

### SIMCA Pattern Recognition

SIMCA pattern recognition was developed by Wold and coworkers [1] for application to classification problems in chemistry. The technique is based on the modeling of chemical classes by disjoint principal component models. Once the class models have been determined, objects are classified by fitting their data to the class models. A standard deviation for each model is calculated from the residuals which represents a class tolerance level around the model in measurement space.

### Data Analysis

In this study an IBM PC-XT microcomputer with 640K memory was used. A modified version of the SIMCA program was used for the majority of the pattern recognition calculations. The low resolution mass spectra of 78 compounds were obtained from the EPA-NIH Mass Spectral Library on an INCOS data system (see table 1). The GC-MS data files were from routine calibration and analysis performed by the Environmental Monitoring Systems Laboratory, U.S. Environmental Protection Agency, Research Triangle Park, NC.

The data were preprocessed by taking the autocorrelation transform of the mass spectra for mass lags less than 100. In the initial stages of class modeling, the autocorrelation transformed spectra for the 78 training compounds were examined for class separation with two and three dimensional principal components projections of the training data. Three different groups were found: nonhalogenated benzenes; chloroaromatics, chloroalkanes, chloroalkenes; and bromocarbons.

Principal components models were then derived for each class [2]. None of the class models required more than three principal components. The largest number of autocorrelation coefficients relevant to a specific class was 40 in class 3. Eighty-six percent of the training set compounds were assigned to their correct class as a first choice and were within two standard deviations of the models.

### Hierarchical Classification Scheme [2]

Once a class assignment is made, further identification of each spectrum can be made. This is done by calculating the distance, using the entire spectrum in autocorrelation space, of each compound to its three nearest neighbors in its assigned class. To obtain a specific structural assignment, the normalized correlation coefficient of the unknown mass spectrum with that of each of its three nearest neighbors is obtained. If the unknown is not identified as one of the three nearest neighbors in the first class assignment, the correlation coefficients of the three nearest neighbors in the second SIMCA class assignment are compared.

## Results

### GC-MS Calibration Samples

Three direct injection calibration samples, which contained all but one of the training set compounds, were analyzed [2]. Of the 77 compounds observed in the calibration runs, 84% were correctly identified. Of the 13 compounds not identified, three were correctly assigned by chemical class.

### GC-MS Field Samples

The GC-MS data files for three field samples were also analyzed [3]. The identities of the target compounds were determined by using both GC retention times and a combination of forward and reverse spectral matching techniques with stringent matching parameters. The identification of other compounds not on the target list was based on a Finnigan search technique. The application of the pattern recognition scheme to the transformed data for the target compounds resulted in 88% correct classification. The compound identification results were 85% accurate.

There were 75 different nontarget compounds identified in 120 occurrences in the three samples. The classification results agreed very well for the two class 1 and class 2 spectra. However, a very large number of alkanes and alkenes were incorrectly classified as chlorocompounds. Further details of this study are given in references [2], [3], and [4].

Although the research described in this article has been funded by the U.S. Environmental Protection Agency under Cooperative Agreement CR-811617 with the University of Illinois at Chicago, it has not been subjected to Agency review. The mention of commercial products does not constitute endorsement or recommendation for use.

## Figures and Tables

**Table 1:** Compounds included in this pattern recognition study

Compound	Class^a^	Compound	Class^a^
		Dibromomethane	3
		Bromoform	3
*p*-Xylene	1	1,2-Dichloroethane	2
1,3,5-Trimethylbenzene	1	1,1,1-Trichloroethane	2
Isopropylbenzene	1	1,1,2-Trichloroethane	2
*n*-butylbenzene	1	1,1,2,2-Tetrachloroethane	2
1-Methyl-4-isopropylbenzene	1	Pentachloroethane	2
*o*-Dichlorobenzene	2	1,1-Dichloroethane	2
*p*-Dichlorobenzene	2	Trichloroethene	2
l-Chloro-2-methylbenzene	2	Tetrachloroethene	2
l-Chloro-4-methylbenzene	2	Bromoethane	3
*p*-Chlorostyrene	2	1,2-Dibromoethane	3
1,1-Dichloroethane	2	1-Chloropropane	2
1,1,1,2-Tetrachloroethane	2	2-Chloropropane	2
1,2,3-Trichloropropane	2	1,2-Dichloropropane	2
3-Chloro-1-propene	2	1,3-Dichloropropane	2
2-Chlorobutane	2	1-Bromo-3-chloropropane	3
1,3-Dichlorobutane	2	1,2-Dibromopropane	3
1,4-Dichlorobutane	2	2,3-Dichlorobutane	2
l,4-Dichloro-2-butene (*cis*)	2	Tetrahydrofuran	0
3,4-Dichlorobut-1-ene	*2*	Benzaldehyde	1
1,4-Dioxane	0	1-Bromo-1-chloroethane	3
1-Chloro-2,3-epoxypropane	2	2,2-Dibromopropane	3
2-Chloroethoxyethene	2	2-Bromopropene	3
Acetophenone	1	2-Bromopropane	3
Benzonitrile	1	3-Bromopropene	3
Benzene	1	1-Bromopropane	3
Toluene	1	1-Chlorobutane	2
*o*-Xylene	1	1-Bromo-2-chloroethane	3
*m*-Xylene	1	Bromodichloromethane	3
Ethylbenzene	1	1-Bromobutane	3
Styrene	1	2,2-Dichlorobutane	2
Chlorobenzene	2	Dibromochloromethane	3
Bromobenzene	3	1,1,2-Trichloropropane	2
*m*-Dichlorobenzene	2	1,3-Dibromopropane	3
1-Chloro-3-methylbenzene	2	1,1,1,2-Tetrachloropropane	2
Chloroform	2	1,2,2,3-Tetrachloropropane	2
Carbon tetrachloride	2	1,3-Dibromobutane	3
Bromochloromethane	3	1,1,2,3-Tetrachloropropane	2
Bromotrichloromethane	3	1,4-Dibromobutane	3

a Class 1 = nonhaloaromatics; class 2 = chlorocarbons including aromatics and aliphatics; class 3 = bromo- and bromochlorocarbons; class 0 = internal standards and dioxane and tetrahydrofuran.
